# Enhanced cytotoxic effect of doxorubicin conjugated gold nanoparticles on breast cancer model

**DOI:** 10.1186/s13065-022-00889-9

**Published:** 2022-11-09

**Authors:** Amna H. Faid, Samia A. Shouman, Yehia A. Badr, Marwa Sharaky

**Affiliations:** 1 National Institute of Laser Enhanced Science, Cairo University, Giza, Egypt; 2 National Cancer Institute (NCI), Cairo University, Cancer biology, Giza, Egypt

**Keywords:** Gold nanoparticles, Cancer, Drug delivery, Doxorubicin, Breast carcinoma cell line (MCF7)

## Abstract

**Background:**

The difficulty of achieving targeted drug delivery following administration of presently marketed anticancer therapeutics is still a concern. Metallic nanoparticles (NPs) appear to be promising in this regard. The present study focused on the use of gold nanoparticles (AuNPs) as a drug carrier for anticancer Doxorubicin (DOX) forming DOX–AuNPs nanocomposite. The anticancer effect of the prepared nanocomposite was evaluated using SRP essay on breast cancer cell line (MCF7) for different incubation times (24 h,48, and72hr). The prepared DOX–AuNPs nanocomposite was investigated by UV–visible spectroscopy, TEM, fluorescence spectroscopy, and FTIR spectroscopy.

**Results:**

Our results showed that the prepared AuNPs and DOX–AuNPs nanocomposite have spherical and small size10 ± 2 nm and 12 ± 2 nm respectively. The potential cytotoxicity of the DOX-AuNPs nanocomposite on the MCF7 cell line was significantly increased compared to free DOX. The 20 µM DOX- AuNPs nanocomposite produced a similar decrease in cell survival as 80 µM free DOX.

**Conclusion:**

Future work is in progress to investigate the positive effects of the prepared nanocomposite for chemo-photothermal combination treatment.

## Introduction

Cancer is still a major health care problem worldwide. It is estimated that by 2030, there will be 26 million new cancer cases and17 million cancer deaths yearly [1, 2]. The world devotes tens of billions of dollars and huge human capital every year to the development of new anti-tumor drugs. In spite of the tremendous efforts made in the treatment of cancer, it is difficult to achieve a cure rate without damaging healthy cells [3]. A major problem with anticancer drugs involves their toxicity, which can lead to the death of healthy cells and cancer cells [4], and the development of multidrug resistance (MDR) of anticancer drugs [5]. Recently, the emergence of nanotechnology has had a significant impact on clinical treatment. Advances in biocompatible nanoscale drug carriers have made the delivery of anti-cancer drugs more efficient and safer, thereby improving pharmacokinetics and reducing side effects [6]. In cancer treatment, the efficiency of nanoparticles depends on the enhanced permeability and retention effect caused by the leaking tumor vasculature in order to better accumulate drugs at the tumor site [7]. AuNPs have been used as unique drug delivery carriers in clinical applications due to their characteristics such as shape, size, and surface dependence. In addition to its biocompatibility, it is non-cytotoxic and its surface can be easily functionalized [8]. Among various chemotherapy drugs, doxorubicin (DOX) is a drug widely used as an anti-cancer drug. Although it kills cancer cells by inhibiting the synthesis of nucleic acid in the cell, three main problems are encountered with DOX first high toxicity and a large volume of distribution, second, a short lifetime in the body, and third low solubility [9] which results in a narrow therapeutic index. To overcome the non-specificity and high toxicity of doxorubicin many researchers have proposed conjugation to nanoparticulate delivery systems to reduce the toxicity level while sustaining the therapeutic efficacy [10].

In the present study, we report on the synthesis of AuNPs using trisodium citrate as a reducing and stabilizing agent followed by loading DOX to form DOX– AuNPs nanocomposite. The formation process of the prepared nanocomposite composites was investigated via Transmission electron microscope, UV-Visible, Fourier transform infrared and Fluorescence spectroscopy. The results showed that the cytotoxic effect of DOX-AuNPs nanocomposites on MCF7 reduced side effects and enhanced the therapeutic effect compared with an equal dose of free DOX.

## Materials and methods

### Preparation of AuNPs

AuNPs were synthesized according to the standard wet chemical method [11]. Trisodium citrate (38.8 mM, 10 mL) was added to a boiling HAuCl4 solution (1 mM, 100 mL). After addition, the yellow-colored solution of gold chloride turned the wine red in color. The formed particle size and shape were investigated using a TEM and UV-Visible spectrophotometer.

## Preparation of DOX– AuNPs nanocomposite

DOX-AuNPs nanocomposite was made according to *Vaithilingam* method [12]. 1 ml of different concentrations of DOX (10^− 3^, 10^− 4^, 10^− 5^, 10^− 6^ M) were mixed dropwise with 20 ul/ml of AuNPs with continuous stirring and sonicate for 10 min until deep red become blue.

### Characterization of AuNPs and DOX–AuNPs nanocomposite

#### UV-Visible spectrophotometer analysis

UV-visible absorbance spectra were measured using ***a*** double beam spectrophotometer ***(PG instrument, T80***^***+***^, ***UK)***. 200 µl from (AuNPs, 10^− 5^ M free DOX and DOX-AuNPs nanocomposite) were diluted to 2 ml with distilled water then placed in 1 cm UV-quartz and the absorption was recorded within the appropriate scan range (200 to 800 nm). The spectra were taken against distilled water as the pure solvent reference for each sample.

#### Transmission electron microscope analysis

The morphology of the prepared solutions was carried out using TEM – Nanotechnology& Advanced Material Central Lab. (NAMCL), Agriculture Research Center (ARC). Company name: FEI, Netherland. Model: Tecnai G20, Super twin, double tilt, and Applied voltage: 200 kV, Magnification Range: up to 1,000,000 X, and Gun type: LaB6 Gun. A drop from very dilute solutions was deposited on an amorphous carbon-coated copper grid and left to evaporate at room temperature forming a monolayer then detected by TEM.

#### Photoluminescence (PL) spectroscopy analysis

Photoluminescence spectra of 200ul of (free DOX, and DOX-AuNPs) diluted to 2 ml with distilled water were recorded, using UV-quartz cuvettes of 1 × 1 cm^2^, with a *Perkin Elmer LS55* Spectrofluorometer. equipped with a xenon short-arc lamp as an exciting source. The spectra were measured at right angle excitation. The excitation wavelength was 470 nm, the slit width was 10 nm and the scan speed were 500 nm.

#### Fourier transforms infrared (FTIR) spectroscopic analysis

IR measurements were carried out using FT-IR spectrometer (Shimadzu FT-IR 8400) in the range (500 − 4500 cm^− 1^). Prepared samples (free DOX and DOX-AuNPs) were dried using *lypholizer*.IR spectra of powdered samples were diluted with a potassium bromide (KBr) pellet. Where 4-8 mg from the dried samples were added to 200 mg KBr then careful grinding the sample was of great importance for the elimination of errors caused by scattering.

### Cytotoxicity of DOX and DOX-AuNPs on MCF7

This method was carried out according to that of Skehan et al. (1990) [13]. Cells were seeded in 96-well microtiter plates at a concentration of 5 × 10^3^ Cell/well in a fresh medium and left to attach to the plates for 24 h. Cells were incubated with different concentrations of AuNPs (150, 250, 350 and 450ul/ml),free DOX (5 × 10^− 6^, 10^− 5^, 2 × 10^− 5^,4 × 10^− 5^, 8 × 10^− 5^ M) and DOX-AuNPs with the same DOX concentrations on 20 ul/ml AuNPs then completed to a total of 200 µl volume/well using fresh medium and incubation was continued for 24, 48 and 72 h. For each drug concentration, three wells were used. Following 24, 48, and 72 h treatment, the cells were fixed with 50 µl cold 50% trichloroacetic acid for 1 h at 4 0 C. Wells were washed 5 times with distilled water and stained for 30 min at room temperature with 50 µl 0.4% SRB dissolved in 1% acetic acid. The percentage of cell survival was calculated as follows: Survival fraction = O.D. (treated cells)/ O.D. (control cells). The IC_50_ values (the concentrations of thymoquinone required to produce 50% inhibition of cell growth).

### Statistical analysis

Data are expressed as the arithmetic mean ± SD. Statistical analysis was carried out using GraphPad Software Prism v5 (San Diego, USA). The statistical analysis of the transfection assay data was done using Tukey multiple comparison test One-way analysis of variance (ANOVA)with a single pooled variance. Differences were considered statistically significant when p ≤ 0.05.

## Results and discussion

### Characterization of DOX loaded AuNPs (DOX-Au nanocomposite)

**Fig. 1:**
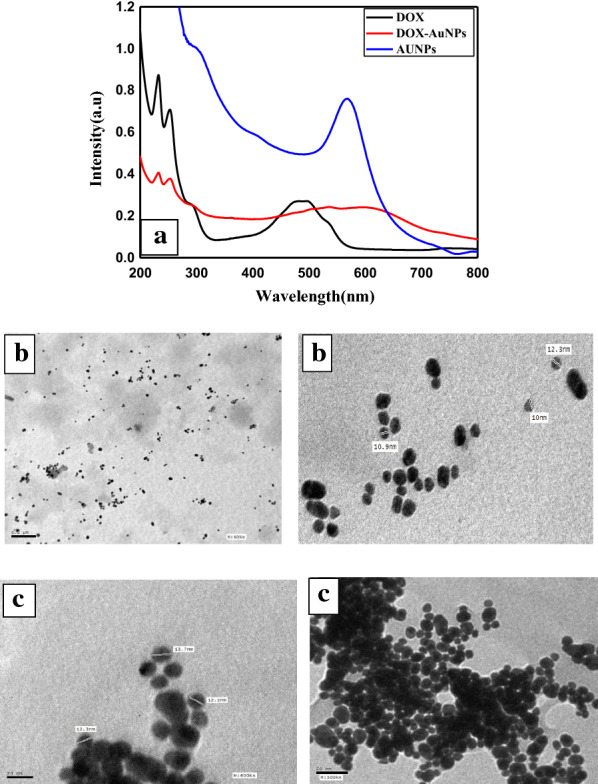
**a **UV- Visible Spectrum of spherical AuNPs, DOX 10^− 5^ M and DOX -AuNPs nanocomposite 10^− 5^ M, **b** TEM images of AuNPs and **c** DOX –AuNPs nanocomposite

As shown in Fig. 1, the prepared AuNPs show absorption in the visible range due to Surface Plasmon Resonance (SPR) at 520 nm [14]. TEM observations were employed to clarify the morphology of formed AuNPs, the particles are spherical with approximate size (10 ± 2 nm) with uniform size distribution Fig. 1b.

**Fig. 2:**
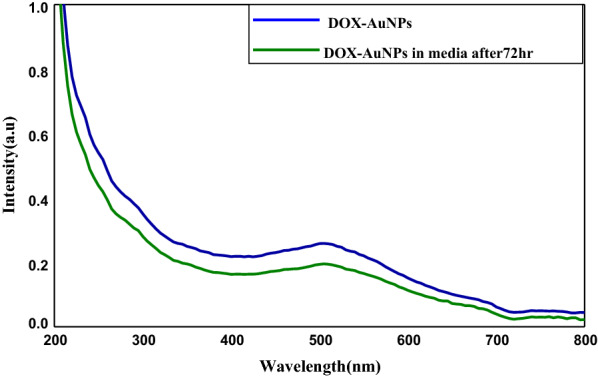
UV- Visible Spectrum of DOX-AuNPs and DOX-AuNPs in media after 72 h

As shown in Fig. 1a, DOX exhibits a visible band at 495 nm characterized by anthracyclines, as well as absorption in the UV range from 200 to 300 nm [15]. Upon addition DOX to AuNPs there were a reduction in band intensity which is accompanied by the emergence of new bands at 630 nm as a result of aggregation owing to interparticle interaction between AuNPs and DOX. The color change from red to violet and a small growth in the AuNPs size is a conformation of aggregation Fig. 1a. The replacement of citrate with DOX to form DOX-AuNPs nanocomposite may be the main reason for AuNPs aggregation. Moreover, the better electrostatic attraction of DOX with AuNPs than citrate groups might be accountable for the extra band at longer wavelengths. It has been revealed theoretically and experimentally that aggregation of AuNPs leads to additional plasmon absorption at higher wavelengths when nanoparticles are electronically attached to each other [16]. Consequently, violet color of the DOX-AuNPs solution as well as the UV-Vis spectrum slightly unchanged after 72 h storage in DMEM at room temperature, indicating the formation of a stable particle suspension, as shown in Fig. 2. TEM pictures of DOX-AuNPs nanocomposite in Fig. 1c; showed that the nanocomposite had a spherical shape and smooth surface with small growths in the particle size from 10 ± 2 nm to 12 ± 2 nm. When coupled particles were arranged in a linear pattern, the plasmon coupling phenomena was plainly visible.

### The effect of DOX concentration

**Fig. 3:**
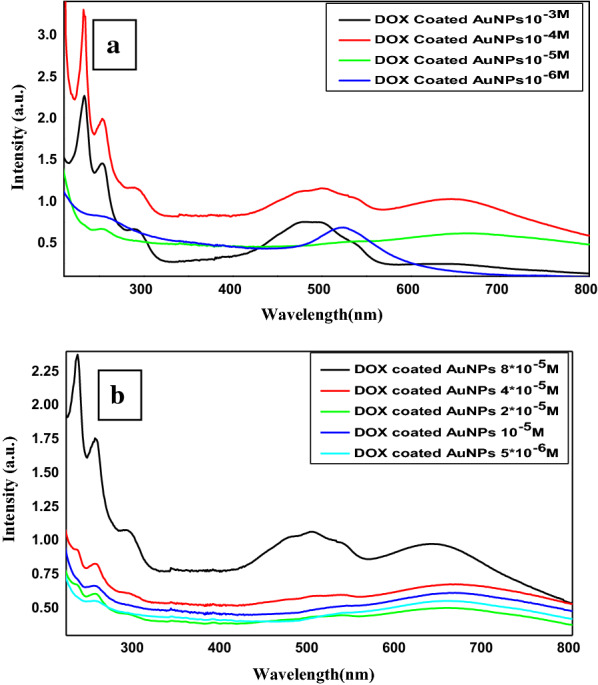
**a** UV-Visible Spectrum of 10^− 3^, 10^− 4^, 10^− 5^ and 10^− 6^ M DOX coated AuNPs, **b** UV Visible Spectrum of 5 × 10^− 6^, 10^− 5^, 2 × 10-^5^, 4 × 10^− 5^and 8 × 10^− 5^ M DOX coated AuNPs

To test for the critical concentration for loading DOX on AuNPs, different concentrations of DOX were prepared (10^− 3^, 10^− 4^, 10^− 5,^ and 10^− 6^ M). As shown in Fig. 3A, a new band at 630 nm which is the characteristic band for coating of DOX on AuNPs has appeared at DOX concentrations 10^− 4^ M and 10^− 5^ M. While at DOX concentration 10^− 3^ M the characteristic bands for free DOX appear which may be corresponding to the load of specific concentration of DOX on AuNPs and there was excess free DOX in the solution. Moreover, at DOX concentration 10^− 6^ M, the absorption band of free AuNPs is the dominant band which may be because this concentration is lower than the critical concentration for loading and free AuNPs are presented in the solution which is the dominant band. To investigate the therapeutic efficiency of DOX- AuNPs nanocomposite different concentrations of DOX loaded AuNPs (5 × 10^− 6^, 10^− 5^, 2 × 10^− 5^, 4 × 10^− 5,^ and 8 × 10^− 5^ M) were prepared and the absorption spectra have been measured. Data cited in Fig. 3B, show that as DOX loaded AuNPs concentration decreased the intensity of the bands at 200 nm to300 nm, 520 and 620 nm decreased and the band at 200 nm slightly disappeared and the band at 630 nm slightly red-shifted with a decrease in DOX loaded AuNPs concentration. These results enable us to choose this scale range for the in vitro study.

### Fluorescence analysis

**Fig. 4:**
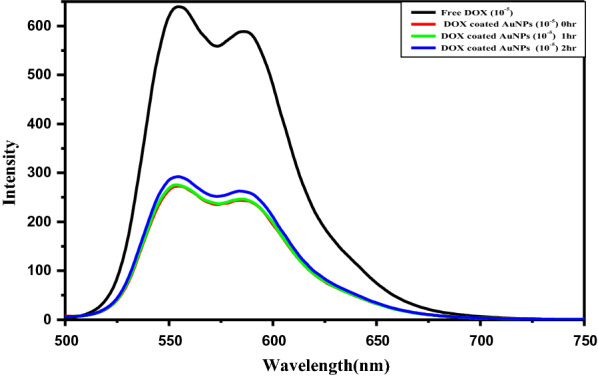
Emission spectra were recorded for free DOX and DOX - AuNPs nanocomposite at various time intervals

Fluorescence studies offer an excellent probe for confirming the binding of drugs with AuNPs as it has been previously reported that gold metal efficiently quenches the emission of many fluorophores. The spectrum recorded is shown in Fig. 4. There was no major change in the spectral profile in DOX loaded AuNPs and the peaks at 550 and 590 nm which are observed for pure DOX were retained in the presence of AuNPs.

The fluorescence intensity was reduced and this quenching of intensity can be attributed to the electronic interactions between DOX and AuNPs due to the binding of the –NH group on AuNPs surfaces, the electronic environment is altered which results in the quenching of fluorescence [12]. However, the preservation of the fluorescence signature supports the claim that DOX structure is retained following complexation with AuNPs; this fact is very important for the biological activity of DOX [17]. As shown in Fig. 4, with increasing the reaction time the fluorescence intensity is drastically quenched. This may be because with an increase in reaction time more amount of DOX gets bound to AuNPs and hence this causes a further decrease in fluorescence intensity.

### Fourier Transform Infra-Red Spectroscopy (FT-IR) measurements

**Fig. 5:**
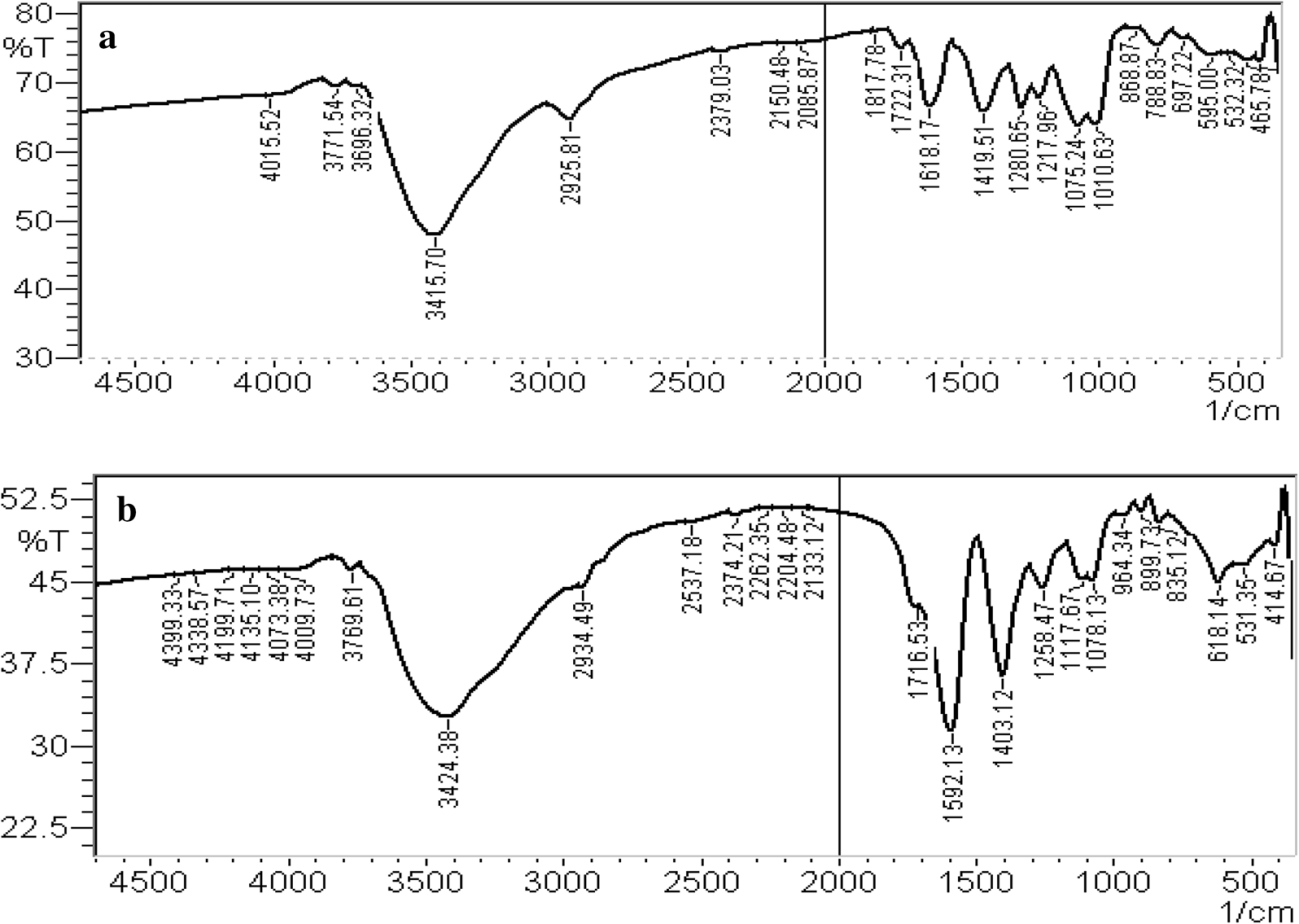
FT-IR transmission spectrum of pure DOX (**A**), DOX - AuNPs (**B**)

The binding interaction of DOX-loaded AuNPs was further investigated using FT-IR studies. The IR spectra of free DOX and DOX-loaded AuNPs are shown in Fig. 5. Pure DOX shows bands at 3415 cm^− 1^, 1217 cm^− 1,^ and 2085 cm^− 1^corresponding to–NH stretching frequency, C-N stretching and C = C stretching respectively Fig. 5 A. In the case of DOX-loaded AuNPs, there were a decrease in intensity of all bands and NH which is now broadened and slightly shifted to higher wavelengths at 3424 cm^− 1^. And there were redshifts for C-N stretching and C = C stretching to 1258 cm^− 1^ and 2133 cm^− 1^ respectively Fig. 5B.

It could be understood that it is the free –NH group that is likely to be involved in the binding of DOX on the AuNPs surface as it is well known that gold has a strong affinity toward amino groups [18]. The results given here were accorded well with Dhar et al. [17].

### Cytotoxic effect of DOX and DOX-AuNPs nanocomposite on cellular proliferation of MCF7 cell line

**Fig. 6:**
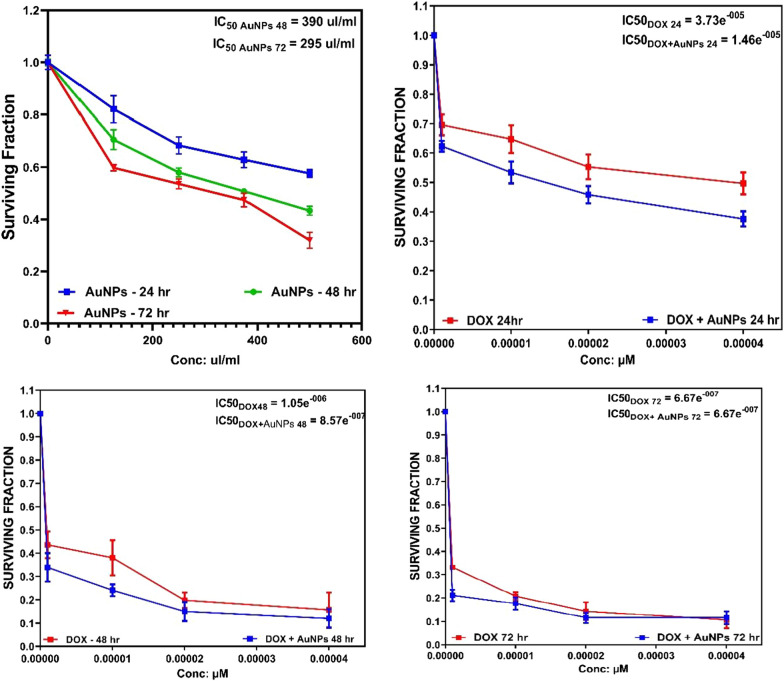
Cytotoxicity test at different concentrations of AuNPs, DOX and DOX-AuNPs nanocomposite on MCF7 cell line following 24, 48, and 72 h

Figure 6, reveals the effect of different concentrations of AuNPs, DOX and DOX- AuNPs nanocomposite (5, 10, 20, 40, and 80 µM) on the percentage of survival of breast carcinoma cell line (MCF7) after 24, 48, and 72 h exposure to the drug. It is to be noted that at each concentration the DOX-AuNPs nanocomposite was taken such that the DOX concentration was similar to that in free solution making a direct comparison possible. At all the times studied there were a concentration and time-dependent decrease in cellular proliferation compared to its respective control. Cytotoxicity is presented as the survival fraction compared to the untreated control cells. The comparisons of IC50 for free DOX and DOX-AuNPs nanocomposite with different concentrations at 24 h, 48 h, and 72 h was shown in Table 1. DOX-AuNPs nanocomposite exhibited a lower *IC50* value of 1.46e^− 5^ M and 8.57e^− 7^ M than free drug where the observed *IC50* values were 3.73e^− 5^ M and 1.05e^− 6^ M in 24 and 48 h respectively. While in 72 h the *IC50* for free DOX and DOX-AuNPs nanocomposite were of the same value of 0.667 µM which was less than 24 and 48 h. Moreover, AuNPs produce a decrease in cell viability reaching *IC50* value of 390 and 295 μl/ml after 48 and 72 h. We use the safe concentration of AuNPs 20 ul/ml as it produces a decrease in cell with 10% for loading DOX.

DOX has been widely used for the treatment of a broad spectrum of cancers. The capacity of doxorubicin to interfere with DNA activity and cause DNA damage is one of the mechanisms through which it exerts its proapoptotic effects. DOX has the ability to cross-link DNA strands, block topoisomerase II, and intercalate into DNA double helices. It is also known that doxorubicin causes the formation of reactive oxygen species (ROS) through a one-electron reduction to the equivalent semiquinone free radicals, which subsequently quickly react with oxygen to form superoxide radical anions [19]. Both DNA damage and an increase in ROS can contribute to doxorubicin-mediated apoptosis. It is possible that the DOX-AuNPs nanocomposite’s cytotoxicity will increase as a result of increased drug accumulation at the site of action. targeted delivery. A possible explanation for the activity enhancement of DOX-AuNPs nanocomposite is the improvement due to the internalization of DOX-AuNPs nanocomposite by an endocytosis mechanism. Generally, nanoparticles are nonspecifically internalized into cells via endocytosis or phagocytosis compared to the passive diffusion mechanism of free DOX into cells [20, 21]. Our result was in accordance with Dhar *et al. 2008* [17]. As shown in Fig. 6 AuNPs showed nearly non-cytotoxic at 20 ul/ml while DOX loaded AuNPs caused a strong decrease in cell viability which indicted that AuNPs under study has good cell viability percentage at the tested concentration for drug loading [22]. This result is similar to our expectation, that the free AuNP has good biocompatibility, and it confirmed an excellent cytotoxicity impact after loading with Dox.

**Table 1:** Comparison of IC50 values for Free DOX and DOX coated AuNPs at 24 h, 48 h, and 72 h

Cell line	IC_50_					
	Free DOX			DOX –AuNPs nanocomposite		
	24 h	48 h	72 h	24 h	48 h	72 h
MCF7	3.73e^− 5^ M	1.05e^− 6^ M	6.67e^− 7^ M	1.46e^− 5^ M	8.57e^− 7^ M	6.67e^− 7^ M

## Conclusion

The present work demonstrated a method for coating DOX on AuNPs forming nanocomposite for breast cancer treatment. The prepared AuNPs and DOX-AuNPs nanocomposite were spherically shaped with an average particle size of 10 ± 2 nm and 12 ± 2 nm respectively. Loading of DOX on AuNPs was found to be dependent on drug concentrations. Then both free DOX and DOX-AuNPs nanocomposites were examined and compared for their anti-proliferation activities against the MCF7 breast cancer cell line. The results showed that Coating DOX on AuNPs significantly enhance the anti-proliferation activity on the MCF7 cell line, Future work is in progress to investigate the positive effects of the prepared nanocomposite for chemo-photothermal combination treatment in vitro and in vivo cancer treatment

## Data Availability

The datasets used and/or analyzed during the current study are available from the corresponding author on reasonable request.

## Abbreviations

AuNPs: Gold nanoparticles
DOX: Doxorubicin
EPR: Enhanced permeability and retention effect
FTIRs: Fourier transform Infrared spectroscopy

## References

[1] Ginsberg G, Lim V, .Johns VLauer,B (2010). C ,Sepulveda. Prevention,screening and treatment of colorectal cancer: a global and regional generalized cost effectiveness analysis. Cost EffResourAlloc.

[2] Torre LA, Bray F, Siegel RL, Ferlay J, Lortet-Tieulent J, Jemal A (2012). Global cancer statistics. CA Cancer J Clin.

[3] .Li SLv,M, .Tang Z, Song W, .Sun H, Liu H (2013). Doxorubicin-loaded amphiphilic polypeptide-basednanoparticles as an efficient drug delivery system for cancer therapy. Actabiomaterialia.

[4] Rabiee N, Yaraki MT, Garakani SM, Garakani SM, Ahmadi S, Lajevardi A, Bagherzadeh M, Rabiee M, Tayebi L, Tahriri M (2020). Recent advances in porphyrin-based nanocomposites for effective targeted imaging and therapy. Biomaterials.

[5] Gottesman MM, Fojo T, Bates SE (2002). Multidrug resistance in cancer: role of ATP-dependent transporters. Nat Rev Cancer.

[6] X.Dong RJ, Mumper (2010). Nanomedicinal strategies to treat multidrug resistant tumors: current progress. Nanomed (Lond).

[7] Zhu J, Tang X, Jia Y, Ho CT, Huang Q (2020). Applications and delivery mechanisms of hyaluronic acid used for topical/transdermal delivery-a review. Int J Pharm.

[8] Gerosa C, Crisponi G, Nurchi VM, Saba L, Cappai R, Cau F, Faa G, Van Eyken P, Scartozzi M, Floris G (2020). Gold nanoparticles: a new golden era in oncology?. Pharmaceuticals.

[9] Robledo-Cadena DX, Gallardo-Pérez JC, Dávila-Borja V, Pacheco-Velázquez SC, Belmont-Díaz JA, Ralph SJ, Blanco- Carpintero BA, Moreno-Sánchez R, Rodríguez-Enríquez S (2020). Non-steroidal anti-inflammatory drugs increase cisplatin, paclitaxel, and doxorubicin efficacy against human cervix cancer cells. Pharmaceuticals.

[10] Schneeweiss A, Möbus V, Tesch H, Hanusch M (2019). Intense dose-dense epirubicin, paclitaxel, cyclophosphamide versus weekly paclitaxel, liposomal doxorubicin (plus carboplatin in triple-negative breast cancer) for neoadjuvant treatment of high-risk early breast cancer (GeparOcto—GBG 84): A randomised phase III trial. Eur J Cancer.

[11] Turkevich J, Stevenso PC, Turkevich JH (1951). A study of the nucleation and growth processes in the synthesis of colloidal gold. Discuss. Faraday Soc.

[12] Selvaraj V, Alagar M (2007). Analytical detection and biological assay of antileukemic drug 5-fluorouracil using gold nanoparticles as probe. Int J Pharm.

[13] Skehan P, Storeng R, Scudiero D, Monks A, McMahon VD, Warren JT, Bokesch H, Kenney S, Boyd MR (1990). New colorimetric cytotoxicity assay for anticancer-drug screening. J Natl Cancer Inst.

[14] Nour M, Hamdy O, Faid AH, Eltayeb EA, Zaky AA (2022). Utilization of gold nanoparticles for the detection of squamous cell carcinoma of the tongue based on laser induced fluorescence and diffuse reflectance characteristics: an in vitro study. Lasers Med Sci..

[15] Ric CH, Ohio C, Raalte JV, Porland O, Mos EC, Ohio C (1988). Method of determing adriamtcin(doxorubicin) or daunomycin in the environment. App No.

[16] Faid AH, Shouman SA, Thabet NA, Badr YA, Sliem MA (2022). Laser enhanced combinatorial chemophotothermal therapy of green synthesis gold nanoparticles loaded with 6mercaptopurine on breast cancer model. J Pharm Innov..

[17] Dhar S, Reddy M, Shiras A, Pokharkar V, Prasad B (2008). Natural gum reduced/stabilized gold nanoparticles for drug delivery formulations. Chem Eur J.

[18] Aslam M, Fu L, Su M, Vijayamohanan K, Dravid PV (2004). Novel one step synthesis of amine-stabilized aqueous colloidal gold nanoparticles. J Mater Chem.

[19] Navarro R, Martinez R, Busnadiego I, Ruiz-Larrea MB, Ruiz-Sanz JI (2006). Doxorubicin-induced MAPK activation in hepatocyte cultures is independent of oxidant damage. Ann N Y Acad Sci.

[20] Faid AH, Shouman SA, Badr YA, Sharaky M (2022). Enhanced photothermal heating and combination therapy of gold nanoparticles on a breast cell model. BMC Chem.

[21] Li L-S, Bin Ren X, Yang Z-CC, Zhao X-J, Zhao M-X (2021). Hyaluronic acid-modified and doxorubicin-loaded gold nanoparticles and evaluation of their bioactivity. Pharmaceuticals.

[22] Kamell S, Faid AH, Hamdy O, Eltayeb EA, Zaky AA (2022). Evaluation of gold nanoparticles for the detection of oral squamous cell carcinoma using Raman spectroscopy: in-vitro study. NeuroQuantology.

